# Comparative *ex vivo* Investigations on the Cutting Quality of the CO_2_ Laser and the Diode Pumped Er:YAG Laser

**DOI:** 10.3389/fsurg.2021.764450

**Published:** 2021-12-14

**Authors:** Holger Wurm, Patrick Johannes Schuler, Florian Hausladen, René Graesslin, Thomas Karl Hoffmann, Karl Stock, Elisabeth Friederike Reins

**Affiliations:** ^1^Institut für Lasertechnologien in der Medizin und Meßtechnik, Ulm University, Ulm, Germany; ^2^Department of Otorhinolaryngology, Head and Neck Surgery, Ulm University Medical Center, Ulm, Germany

**Keywords:** CO_2_ laser, Er:YAG laser, diode pumped, high repetition rate, 2.94 μm, head and neck surgery, oncology

## Abstract

**Objectives:** A sufficient histological evaluation is a key pillar in oncological treatment, especially in situations of cancer of unknown primary. CO2 laser technology is used in clinical routine of soft tissue surgery because of its cutting quality and availability. Diode pumped solid state Er(bium):YAG laser systems promise a higher cutting efficiency and minor thermal damages. The aim of this study was to compare both laser systems with respect to their suitability for cutting soft tissue.

**Methods:** A setup was realized which enables comparable experiments with the clinical CO_2_ laser (AcuPulse 40ST DUO, Lumenis) and the Er:YAG laser system (DPM 40, Pantec Biosolutions AG). Fresh mucosal samples of porcine tongues were used to determine the influence of laser power and sample velocity on cutting depth and thermal damage width for both lasers. In addition, for the Er:YAG laser, the influence of the pulse repetition rate was examined additionally. For analysis, images of histological sections were taken.

**Results:** In all experiments, the Er:YAG laser shows a significantly higher cutting depth (*P* < 0.0001) and less thermal damage width (*P* < 0.0001) than the CO_2_ laser. For example, at an average power of 7.7 W and a sample velocity of 5 mm/s the Er:YAG laser shows a mean cutting depth of 1.1 mm compared to the CO_2_ laser with 500 μm. While the Er:YAG laser shows a mean thermal damage width of 70 μm compared to 120 μm. Furthermore, the Er:YAG enables the adjustment of the cutting depth and thermal damage width by varying the irradiation parameters. A decrease of the repetition rate leads to a reduction of thermal damage. For example, a repetition rate of 100 Hz results in a thermal damage width of 46 μm compared to 87 μm at 800 Hz at an average power of 7.7 W and a cutting velocity = 5 mm/s while a homogenous cutting quality can be achieved.

**Conclusions:** In conclusion, the results of these *ex vivo* experiments demonstrate significant advantages of the diode pumped Er:YAG laser system for soft tissue ablation compared to the CO_2_ laser, in particular regarding cutting efficiency and thermal damage width.

## Introduction

Laser surgery is used in various procedures in head and neck surgery. It has become an alternative to open surgery in the excision of tumors in hard-to-reach regions like the larynx and hypopharynx, reducing the risk of injuring surrounding organs and often preserving their functions (1, 2). Due to the high absorption coefficient of its wavelength (λ = 10.6 μm; μ_a_ = 800 cm^−1^) in water the CO_2_ laser shows a more efficient and precise soft tissue cutting compared to other lasers, working with vaporization (3) and has therefore become the standard in head and neck laser surgery over the past decades (4–7).

While simultaneous coagulation during the ablation process leads to local hemostasis and the reduction of reconstruction needs (1, 8–10), laser irradiation may also cause thermal damage width of the surrounding tissue. The reason for this is the gaussian spatial beam profile and the continuous wave (cw) operating mode which is true for the most of available CO_2_ lasers. Especially when evaluating the infiltrating potential of small lesions or in situations of cancer of unknown primary, it is very important to minimize peripheral damage in order to allow sufficient histological evaluation (11). Furthermore, a prolonged wound healing in comparison to cold surgery has been described (8).

Especially from these points of view the Er:YAG laser shows some decisive advantages. In contrast to the CO_2_ laser, fibers (e.g., sapphire, germanium oxide or ZBLAN fibers) are available for the Er:YAG, which is particularly useful for endoscopic applications. The ablation efficiency of the Erbium laser is even higher compared to the CO_2_ laser (12, 13). The reason is the higher absorption coefficient in water (Er:YAG laser: λ = 2.94 μm, μ_a_ = 1^*^10^4^ cm^−1^, Er:YSGG laser: λ = 2.79 μm, μ_a_ = 4^*^10^3^ cm^−1^) and the pulsed operation mode which leads to higher powers within one laser pulse compared to a cw-system of comparable average power. This leads to so called thermomechanical ablation, in which the massive increase in volume of the water during rapid evaporation results in very efficient tissue ablation (14–16). Furthermore, the more Top-Hat like beam profile, caused by a higher number of laser modes, leads to much steeper temperature gradients at the edges of the sections and thus to significantly fewer thermal side effects such as coagulation and carbonization. But the comparable low repetition rate of the flashlamp pumped Erbium lasers does not allow homogeneous cutting which has left it irrelevant for tumor surgery up to now. The new diode pumped Er:YAG laser system enabling pulse repetition rates up to 2 kHz might eliminate this disadvantage. Furthermore, it offers adjustable pump current as well as a variable pulse duration from 1 to 1,000 μs and offers a better beam quality which allows to couple into fibers with 200 μm core size (11, 17). In prior *in vitro* studies we have already shown that smooth and homogeneous cuts can be achieved in both soft and hard tissue with thermally damaged zones adjustable over a wide range from about 50 μm to > 1,000 μm (18–21).

In this *in vitro* study we compare the cutting characteristics of the new diode pumped Er:YAG laser to a standard clinical CO_2_ laser system using the same clinically approved irradiation parameters on mucosa of the tongue.

## Materials and Methods

### Sample Preparation

The laser cuts were performed on the mucosa of fresh porcine tongues from the slaughter. For this, equivalent tissue samples (thickness = 1 cm) were cut from the lateral part of the tongue. Each parameter setting was performed on 3 different samples. Cutting depth and thermal damage were measured in six different histological sections, respectively (*n* = 18).

### Laser Systems and Experimental Setup

**Figure 1:**
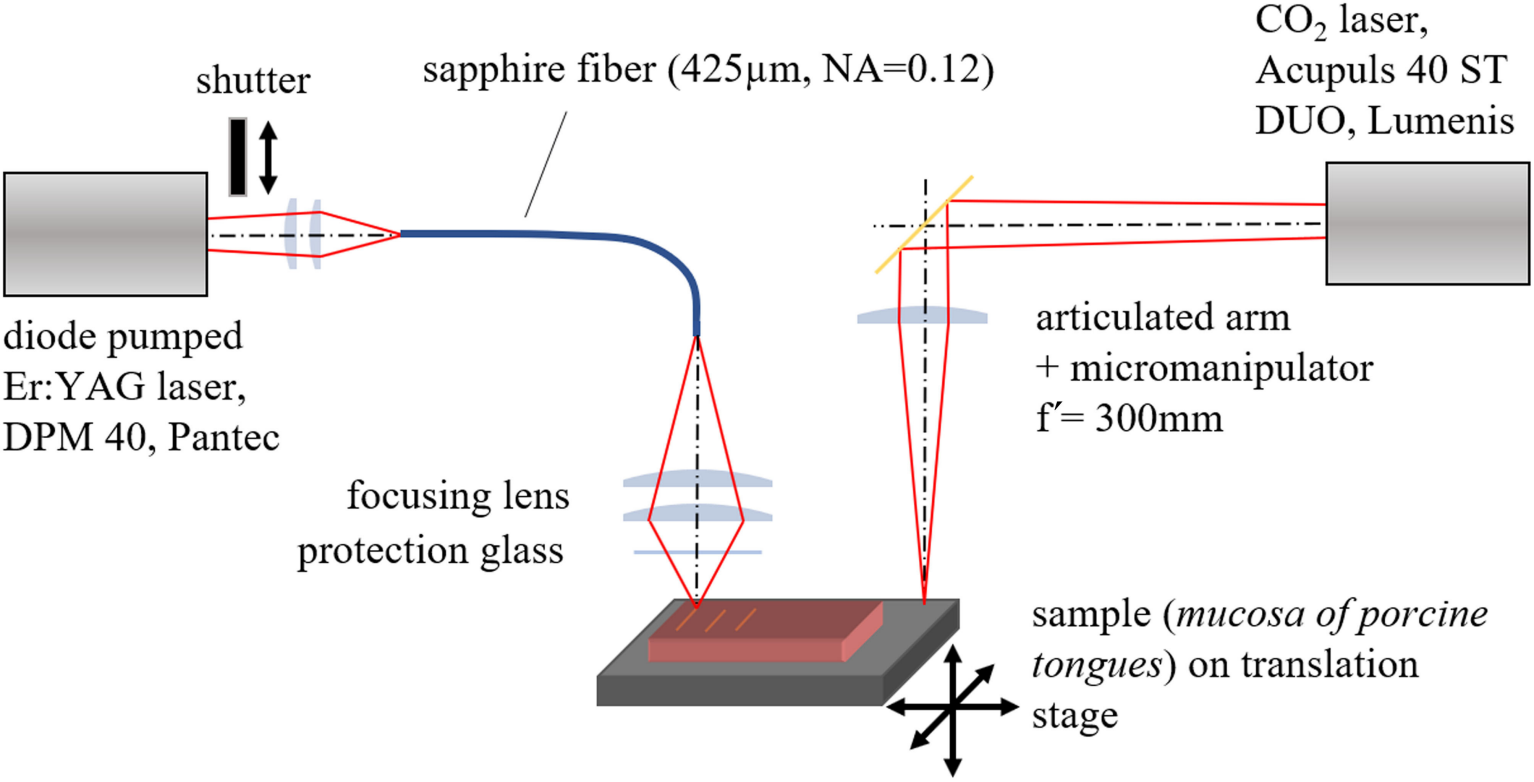
Experimental setup for irradiation of the samples with defined parameters.

Table 1 shows the most important parameters of the used laser systems. The experimental setup is shown in Figure 1. For the CO_2_ laser the standard focusing unit (f = 300 mm; spot diameter = 500 μm) was used. The beam of the Er:YAG laser was coupled into a sapphire fiber (core diameter 425 μm, NA = 0.12) and the fiber output end was imaged onto the sample surface by a specially raytracing designed optic (f' = 66.2 mm) (OpticStudio 20, Zemax) which leads to an almost homogeneous irradiated circular area (diameter = 500 μm) in the image plane of the optics. The measured depth of focus was about 5 mm. A mirror joint arm connects the CO_2_ laser with the irradiation optics (f' = 300 mm), which forms a Gaussian beam waist in the focal plane. The irradiation spot of the Er:YAG laser and the beam waist of the CO_2_ laser were positioned next to each other in a distance of 100 mm in order to allow a reliable switching between the lasers using the same translation stage. The exact size and the position of the image / focus plane were analyzed by moving irradiated photographic paper (burn paper) through the image / focus plane region with a computer-controlled translation stage (Corvus Eco & 3xLS110, Pi miCos GmbH) at a speed of 30 mm/s and low pulse repetition rate. Therefore, the burn paper was moved 10 mm in x- and z-direction simultaneously with equal velocities. The ablation marks of the laser pulses on the paper were analyzed with microscope and the correct positions saved in the control software of the translation stage. The sample was positioned on a holder adapted to the translation stage which allows a defined positioning and movement of the sample during irradiation. After determination of the position all cuts were performed with the translation stage in the image plane / focal plane.

**Table 1:** Parameters of the used laser systems.

	**CO_**2**_ laser**	**Diode pumped Er:YAG laser**
Type	AcuPulse 40ST DUO	DPM40
Manufacturer	Lumenis	Pantec
Wavelength	10.6 μm	2.94 μm
Max. optical power Φ_max_	40 W	40 W
Operation mode	SuperPulse	Pulsed (50 Hz−2 kHz)
Beam quality factor M^2^	≈ 1 (TEM00)	≈25
Focal length f'	300 mm	66.2 mm
Beam waist / Spot diameter	500 μm	500 μm

By using a computer-controlled shutter unit in the beam path of the Er:YAG laser it was possible to reproduce the same procedure for each sample: (a) switching the laser on, (b) waiting for about 10 s to stabilize the laser operation, (c) starting the movement of the sample, (d) opening the shutter automatically when constant sample velocity is reached. After one cycle with a sample movement of typically 10 mm the shutter was closed and the sample stopped. The procedure for the CO_2_ laser was performed in a similar way, using the foot switch instead of the computer-controlled shutter, which leads to an inaccuracy at the beginning and end of the cut. For this reason, the histological sections were taken from the middle part of the cut. The setup is shown in Figure 1.

Prior to the experiments the laser power of the CO_2_ laser was set to 10 W which is a typically used value for soft tissue cutting in a clinical setting. The resulting laser power in the beam waist (7.7 W) was measured by a power meter (30(150)A, OPHIR and Nova II, OPHIR) and this value was also used for the Er:YAG laser. To adjust the laser power the pulse peak current of the Er:YAG laser was kept constant (300 A) and the pulse duration was varied.

### Analysis of the Samples

During the irradiation, the cutting process was recorded by a CMOS-sensor camera (MQ042CG-CM, software XIMEA Cam Tool, XIMEA GmbH) adapted on a surgical microscope (OPMI 6-CFC on Universal S3 stand, Carl Zeiss).

**Figure 2:**
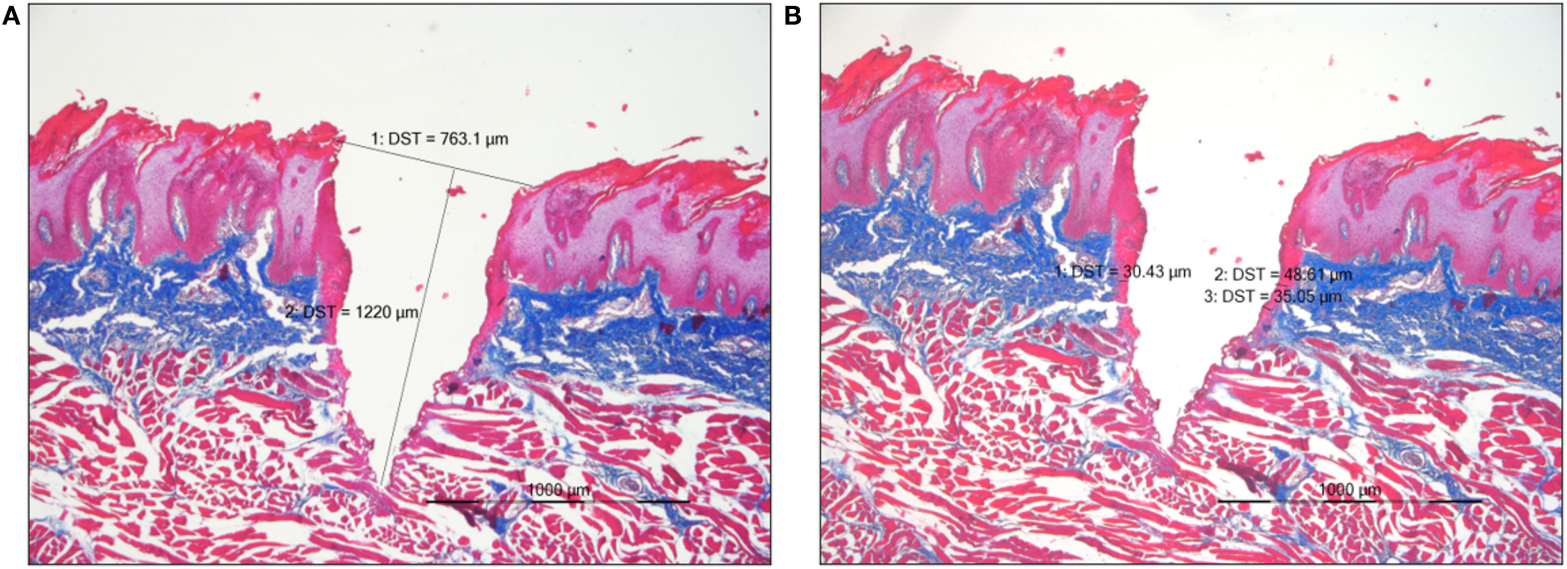
**(A)** Measurement of the cutting depth in a histological section of pork tongue mucosa. Er:YAG, Azan staining, light microscopy at a magnification of 20x. **(B)** Measurement of the thermal damage width in a histological section of pork tongue mucosa. Er:YAG, Azan staining, light microscopy at a magnification of 20x.

To analyze the cutting geometry and to maintain the tissue structure the samples were stored in 4 % formalin solution (neutral buffered formalin) for 72 h for fixation. After embedding in paraffin, the histological sections were prepared and stained with Azan. For image acquisition and evaluation a light microscope (Axiophot, Carl Zeiss) equipped with a digital camera (ProgRes C12plus, Jenoptic) with capture and processing software (Jenoptic, ProgRes Capture Pro, Version 2.5) was used. This software also allowed to measure the thermal damage and the depth of the cuts as shown in Figure 2. The measured thermal damage width includes coagulation and carbonization.

### Statistical Analysis

**Figure 3:**
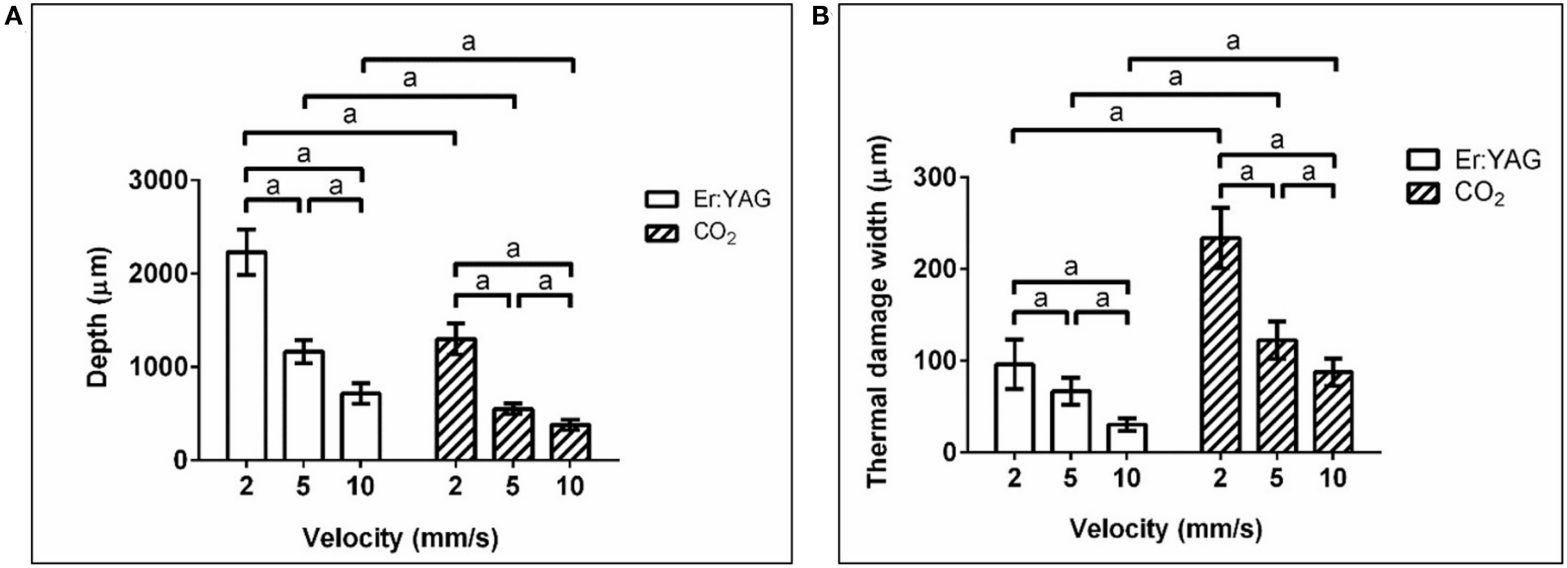
**(A)** Mean value and standard deviation of the measured cutting depths (^*a*^*P* < 0.0001) and **(B)** thermal damage width (^*a*^*P* < 0.0001) for the Er:YAG laser with 200 Hz repetition rate and the CO_2_ laser at various cutting velocity (laser power = 7.7 W), measured in the histological sections.

Statistical analysis was performed using GraphPad Prism six software (GraphPad Software). Data was tested for normal distribution using D'Agostino–Pearson omnibus normality test. Parametric data from Figure 3 was analyzed using the two-way analysis of variance (ANOVA) to determine differences between two grouping variables. Parametric data from **Figure 5** was evaluated using the one-way ANOVA to determine differences between the three velocity groups for the same mean power. Significance was set at *p* < 0.05.

### Experiments

The following investigations were performed:

- Comparative experiments with a laser power of 7.7 W and various cutting velocities (2, 5, 10 mm/s). Er:YAG laser parameter: repetition rate = 200 Hz, pulse duration = 154 μs.
- Investigation of the influence of repetition rate (100, 200, 400 and 800 Hz) on to the cutting depth and thermal damage width for the Er:YAG laser (laser power = 7.7 W).
- Investigation of the influence of laser power on to the cutting depth and thermal damage width for the Er:YAG laser at various cutting velocities (2, 5, and 10 mm/s) at a repetition rate of 200 Hz.

## Results

### Comparison of CO_2_ Laser and Er:YAG Laser

While performing the experiments it was observed that the homogeneity of the cuts of both laser systems is comparable. While the cut made by the CO_2_ laser shows thermal damage up to carbonization, the cut of the Er:YAG laser doesn't. Immediately after cutting, the cutting walls collapsed to a certain extent. The corresponding histological section showed that the cut of the CO_2_ laser is broader and minor deep compared to the Er:YAG laser. The thermal damage in the histological section of the CO_2_ laser is more pronounced compared to the Er:YAG laser and vacuoles as well as carbonization are visible at the edges of the CO_2_ laser cuts.

Figure 3 shows the mean values and standard deviations of the cutting depth (left) and the thermal damage width (right) for both lasers and the various cutting velocities. For both lasers the cutting depth increases with decreasing cutting velocity. The cuts generated by the Er:YAG laser are about two times deeper compared to the CO_2_ laser cuts.

Furthermore, it can be seen that the thermal damage width for the CO_2_ laser cuts is at least twice as wide as for the Er:YAG laser cuts at all speeds.

### Influence of the Repetition Rate on the Cutting Depth and Thermal Damage Width for the Er:YAG Laser

**Figure 4:**
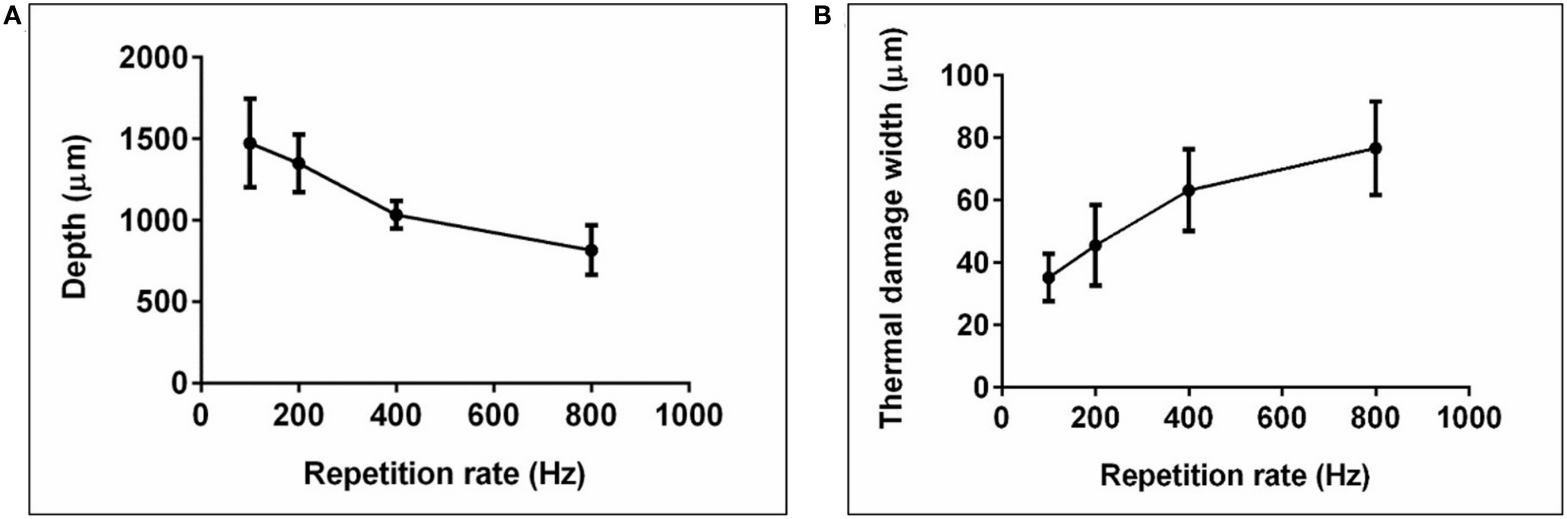
**(A)** Mean value and standard deviation of the measured cutting depth and **(B)** thermal damage width, applied over the repetition rate (Er:YAG laser, laser power = 7.7 W, cutting velocity = 5 mm/s).

Figure 4 shows the resulting mean values and standard deviations of the cutting depth (A) and thermal damage width (B), depicted over the repetition rate. With increasing repetition rate, the cutting depth decreases while the thermal damage width increases.

### Influence of the Laser Power on the Cut Depth and Thermal Damage at Various Cutting Velocities for the Er:YAG Laser

**Figure 5:**
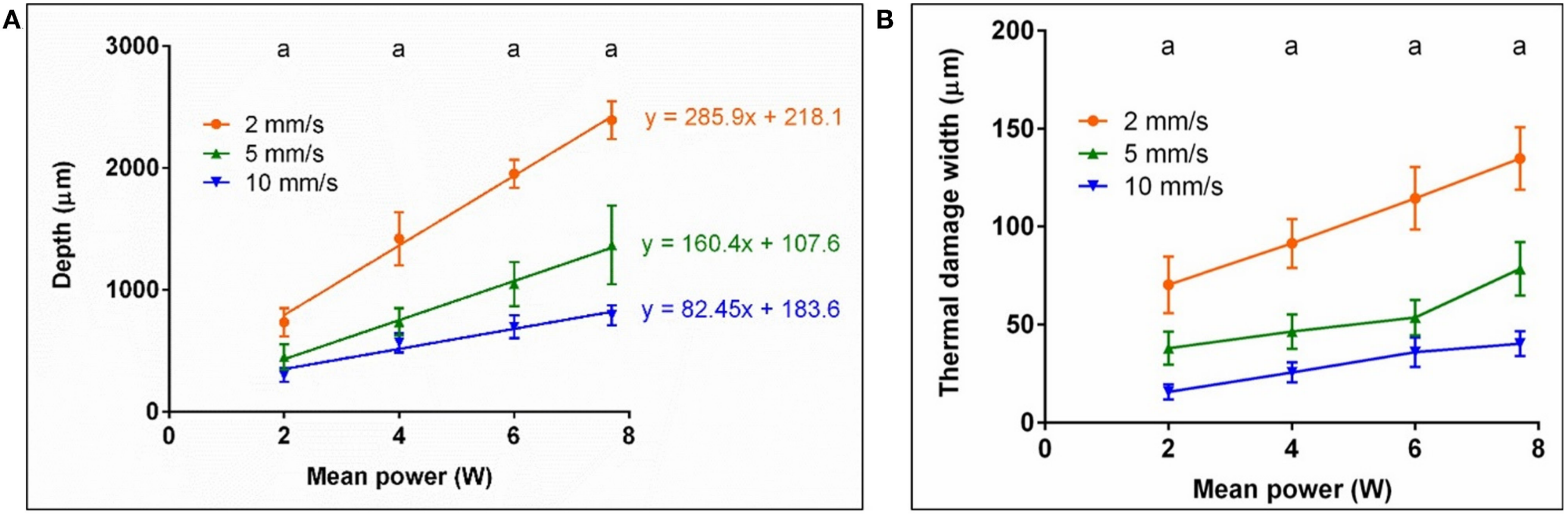
**(A)** Mean value and standard deviation of the measured cutting depths (^*a*^*P* < 0.0001, significant differences between velocities, for the same mean power) and **(B)** thermal damage widths (^*a*^*P* < 0.0001, significant differences between velocities, for the same mean power) applied over the laser power and for various cutting velocities (Er:YAG laser, repetition rate = 200 Hz); in **(A)** linear regressions are shown for the different velocities, including the corresponding equation.

In Figure 5, the resulted mean values and standard deviations of the cutting depth (A) and thermal damage width (B) are depicted over the laser power. Both the cutting depth and the thermal damage width increase with laser power. An increase of the cutting velocity leads to a decrease of both measured values. Especially the cutting depth shows an almost linear behavior with increasing laser power.

## Discussion

In this *in vitro* study on mucosa of fresh porcine tongues we were able to achieve a higher cutting depth (factor ≈ 2) as well as a less pronounced thermal damage of surrounding tissue at comparable homogeneity using the diode pumped Er:YAG laser compared to a standard clinical CO_2_ laser system at clinically approved irradiation parameters.

Both results were at least qualitatively expected due to the higher absorption of the Er:YAG laser radiation in soft tissue and the highly efficient thermomechanical ablation mechanism compared to vaporization. This and the more Top-Hat shaped beam profile in the image plane explain the lack of carbonization when using the diode pumped Er:YAG laser which is expected to be beneficial in terms of better wound healing (8).

The parameters velocity, repetition rate and mean power (pulse energy) have a significant influence on the thermal damage and cutting depth and will be discussed in the following.

The observed decline of cutting depth with increasing cutting velocity (Figure 3) can be explained by the reduced irradiation time per position and therefore a decrease of applied laser energy. This decrease of applied energy per position subsequently leads to the observed reduction of the thermal damage width with increasing cutting velocity.

It is already well known, that at constant mean laser power with rising pulse repetition rates the cutting depth decreases and the thermal damage increases. This can be explained by the increase in the number of pulses per position with increasing repetition rate. The energy to reach the ablation threshold must be introduced into the tissue for each pulse, which leads to increased outflow of energy into the surrounding tissue with a higher number of pulses and thus to the observed increase in thermal damage width and a decrease in cutting depth (Figure 4).

In Figure 5, for all cutting velocities an almost linear correlation between the mean power (and therefore the pulse energy) and the depth of the cuts can be observed. The irradiation time per position follows the equation:


(1) $$\begin{array}{l} t=\frac{\emptyset_{F}}{v} \end{array}$$

with the spot diameter Ø_*F*_ and the cutting velocity *v*. Assuming that the cutting width is equal to the laser spot diameter, the ablation volume Δ*V* can be calculated from the irradiated Area *A* and Δ*z* as follows:


(2) $$\begin{array}{l} \Delta V=A•\Delta z=\frac{1}{4}•{\emptyset_{F}}^{2}•\Delta z\; \end{array}$$

From *m* and *t*, the necessary ablation energy per volume Δ*E*/Δ*V* can be calculated by:


(3) $$\begin{array}{l} \frac{\Delta E}{\Delta V}=\frac{\Delta P•t}{A•\Delta z}=\frac{t}{A•m}=\frac{\emptyset_{F}}{v•A•m}=\frac{\emptyset_{F}}{v•\frac{1}{4}•{\emptyset_{F}}^{2}•m}\\ =\frac{1}{\frac{1}{4}•\emptyset_{F}•m•v}\; \end{array}$$

**Figure 6:**
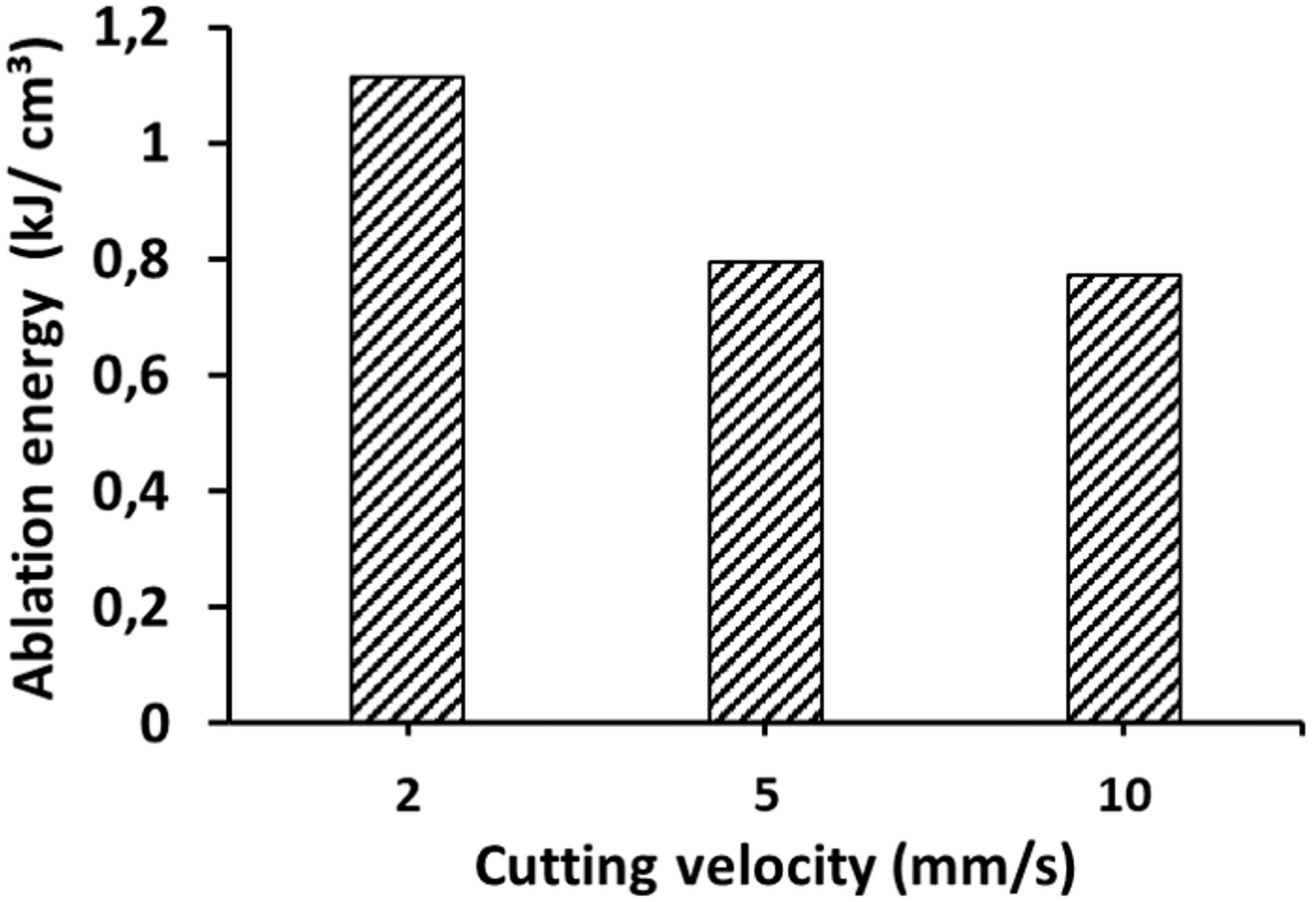
Ablation energy at various cutting velocities, calculated from the slope m of the fitted regression lines in Figure 5 and using Equation 3.

Figure 6 shows the calculated values Δ*E*/Δ*V* for the various cutting velocities. The observed decrease of the ablation energy with increasing cutting velocity can be explained (in a similar manner as above) by the decrease in the number of pulses per position with increasing cutting velocity. All calculated values for the ablation energy are significantly lower than the values found in literature (1.5–5 kJ/cm^2^) (22–25). One possible reason for this could be the top-hat-like beam profile in our experiment.

We were able to achieve homogenous cuts with increased cutting depth compared to the CO_2_ laser. From the perspective of a surgeon the assessment of the right cutting depth requires experience, like with every new tool, to avoid injuring underlying structures. A more effective laser cutting tool, however, might shorten the operation time and thus offer an economic advantage that needs to be evaluated.

Histological studies on the thermal damage width of the CO_2_ laser cut showed the obliteration of small vessels that allows simultaneous hemostasis. The authors also emphasized the lack of reconstruction needs by creating a sealed wound bed (8). Often open surgery with the risk of injuring surrounding organs is the only surgical alternative when trying to reach structures like the laryngopharynx. The thermal damage, however, prevented migration of inflammatory cells as well as spouting of new capillaries and therefore delayed wound healing by several days (26).

A smaller thermal damage width could lead to a higher rate of postoperative bleeding events. On the other hand, a thinner necrotic area could contain scaring, lead to a faster restitution of organ function and therefore prevent the delay of adjuvant therapy.

A thinner thermal damage width could also be a benefit when evaluating the margins of histological samples. If squamous cell carcinoma of unknown primary in the head and neck first presents as cervical lymph node metastases laser surgery is frequently used to take systematic samples from the mucosa of the oropharynx. The management of unknown primary must always include at least a bilateral tonsillectomy and a mucosectomy of the tongue base (27). Primary cancer cell nests can be very small and are not always detectable via positron emission tomography beforehand, but prognosis is significantly better if the primary can be located (28). In this case it is very important to minimize peripheral damage in order to allow sufficient histological evaluation (11).

In conclusion, these experiments demonstrate a higher ablation efficiency with significantly reduced thermal damage and without carbonization. Furthermore, the expand of the thermal damage width can be varied via the repetition rate. Due to the high repetition rates of over 100 Hz and by that the high overlap of the individual pulses, clear cutting edges can be achieved even at high velocities. In combination with the already shown excellent suitability for hard tissue ablation, for example used in stapedotomy, we see a high potential for developing a unique clinical system based on the diode pumped Er:YAG laser. A configuration with similar properties to the CO_2_ laser systems available on the market could be achieved by using a fiber with the smallest possible core diameter and small NA (Numerical Aperture), the end of which would then be imaged onto the tissue on the surgical microscope via suitable imaging optics and an adapted micromanipulator. Furthermore, this configuration would also be very well suited to integrate a therapeutic feedback system, for example OCT or temperature measurement systems, as already described in the literature (29–31). *In vivo* experiments need to be prepared to assess hemostasis, scarring and histological evaluation as well as patient comfort regarding pain and inflammation.

## Data Availability Statement

The raw data supporting the conclusions of this article will be made available on request by the authors, without undue reservation.

## Author Contributions

HW, FH, and KS concepted and developed the optical design for the Er:YAG laser. TH and RG were planning and organizing the experiments with the clinical laser. HW, FH, and ER realized the setup for both laser systems. HW and ER were performing the experiments. HW, ER, and PS were analyzing the histological sections. HW and FH were performing the statistical analysis. HW, KS, ER, and PS wrote sections of the manuscript. All authors contributed to manuscript revision, read, and approved the submitted version.

## Conflict of Interest

The experiments were partially financially supported by Pantec Biosolutions AG. The authors declare that the research was conducted in the absence of any commercial or financial relationships that could be construed as a potential conflict of interest.

## Publisher's Note

All claims expressed in this article are solely those of the authors and do not necessarily represent those of their affiliated organizations, or those of the publisher, the editors and the reviewers. Any product that may be evaluated in this article, or claim that may be made by its manufacturer, is not guaranteed or endorsed by the publisher.
